# Genetic structure of some candidate genes of repeat breeder syndrome in Egyptian buffaloes

**DOI:** 10.1186/s43141-022-00397-2

**Published:** 2022-07-22

**Authors:** Karima F. Mahrous, Heba A. M. Abd El -Kader, Mohamad A. Abdelhafez, Mohamad M. Aboelenin, Esraa A. Balabel, Dalia M. Mabrouk, Osama M. EL Malky, Mohamed S. Hassanane

**Affiliations:** 1 Cell Biology Department, National Research Centre, Giza, Egypt; 2 Animal Production Research Institute, Agricultural Research Center, and Ministry of Agriculture, Dokki, Egypt

**Keywords:** Leptin, Leptin receptor, *BMP*4, Gene polymorphism, Repeat breeding, Egyptian buffaloes

## Abstract

**Background:**

This study aimed to explore the association between polymorphisms in three genes: leptin (*LEP*), leptin receptor (*LEPR*), and *BMP*4, and incidence of repeat breeding in Egyptian buffaloes.

**Methods:**

DNA was extracted from 160 female buffaloes, involving 108 fertile and 52 repeat breeders. Genotyping was performed by polymerase chain reaction-restriction fragment length polymorphism (PCR-RFLP). Sequence analysis and alignment were performed by employing NCBI/BLAST/blastn suite, to identify SNPs among different patterns and alleles. We utilized PredictSNP software to predict the non-synonymous SNPs influences on protein function. Moreover, the conservation score of the amino acids within the target proteins was computed by ConSurf server.

**Results:**

The genotyping results showed that *LEP* and *BMP*4 genes were monomorphic (CC, GG) in all tested fertile and repeat breeder buffaloes. Leptin gene sequencing showed a non-synonymous C73T SNP, replacing R to C at position 25 within the leptin polypeptide (position 4 in the mature form; R4C) which is a neutral mutation, not affecting function or structure of LEP protein. For *LEPR*, one synonymous SNP (T102C) and two non-synonymous SNPs (A106G and C146A), triggering V967A and G954C replacements, respectively in *LEPR* protein. Moreover, they are neutral mutations. Sequencing results of *BMP*4 showed *Hinf*I restriction site indicate fixed GG genotype (CC genotype in the anti-sense strand) in all sequenced samples. No SNPs were observed within the amplified region.

**Conclusion:**

Genotyping and sequencing results of the surveyed three genes revealed that there is no association between these genes mutations and the incidence of repeat breeding in Egyptian buffaloes.

**Supplementary Information:**

The online version contains supplementary material available at 10.1186/s43141-022-00397-2.

## Background

Reproductive features, particularly in monotocous livestock such as cattle and buffalo, are economically significant for long-term food production [1]. An extended period between two calvings (repeat breeding), might be regarded as low reproductive capacity or infertility. This condition necessitates more inseminations, veterinarian attention, and hormonal treatments, all of which affect present and future lactations [2]. Moreover, additional costs are also incurred, because of culling and replacing animals with fertility issues [3].

Fertility improvement is the best choice for lowering culling costs, preserving important genetic traits, and increasing farm profit [4]. According to previous studies, reproductive traits were categorized into binary, interval, and continuous traits with regard to statistical distribution [5]. Ovulation, mating, and calving-related features have been categorized to make reproductive traits easier to comprehend and use in livestock and breeding programs [6].

Leptin (a product of obese *ob* gene), is a versatile 16.4-KDa peptide hormone released primarily by adipocytes, has an important function in reproduction, in addition to regulating body weight and energy expenditure [7]. The bovine *LEP* gene comprises three exons and two introns that spans approximately 18.9 kb, with the coding region of 501 nucleotide length, enclosed in exons 2 and 3, with the first exon not translated into protein [8]. In buffalo, *LEP* has been mapped on chromosome 8 (BBU8q32) [9]. Human, mouse, rat, rabbit, pig, cattle, and buffalo ovaries have all been found to express leptin and its receptors. This provides clear evidence for leptin’s direct engagement in ovarian activities, which runs counter to the popular belief that leptin’s main impacts are on the neuroendocrine component of reproduction [10].

Former studies by Brickell et al. [11] investigated the association of three previously identified single nucleotide polymorphisms (SNPs); exon 2FB, UASMS1, and UASMS2; in *LEP* gene with perinatal mortality (stillbirths and mortality within 24 h of parturition) in 385 British Holstein-Friesian heifers, at first calving. Using RFLP-*Hph*I, Yazdani et al. [12] investigated the effect of the A59V variant in leptin protein in 255 Iranian Holstein cows, finding that the AA genotype had significantly longer pregnancy length than the AB and BB genotypes. Similarly, Clempson et al. [13] examined A59V replacement in leptin protein in 509 Holstein Friesian heifers and found that the CC genotype heifers were younger at the first service and first calving ages. From the same point of view, Komisarek and Antkowiak [14] found a link between fertility and the A59V SNP, in comparison to the CC and CT genotypes, the TT genotype had a shorter day’s open and calving interval, as well as a smaller number of inseminations per conception.

The effects of leptin are mediated by six different leptin receptor isoforms. The leptin receptor is a glycoprotein that has only one transmembrane region. The *LEPR* gene is found on chromosome 3 of bovines. The leptin receptor gene is made up of 20 exons spread out over 1.75 Mb [15]. The long, fully active isoform (*LEPR-b*) is mostly expressed in the hypothalamus, where it plays a role in energy balance and secretory organ activity regulation; nevertheless, it has the potential to act in a variety of peripheral tissues, including gonadal tissues [16]. Leptin receptors are found in the ovary, according to Fu et al. [17], where leptin can control steroidogenesis and improve the ability of the oocyte to maintain subsequent embryonic development.

Many investigations on the effects of leptin and leptin receptor genes on various cattle breeds were conducted. It was confirmed that the SNP *LEPR*/T945M has an effect on milk production traits [18], reproduction traits [13], and growth traits [19]. In cows, selecting animals with the *LEPR*/T945M gene could improve productivity and reproduction qualities.

Bone morphogenetic proteins (BMPs) are candidate genes belonging to the member of the TGF-β (Transforming Growth Factor-beta) superfamily. So far, more than 30 members have known in BMP family, of which *BMP4* is the prime [20]. As it plays a key role in the development and growth of both the ovarian follicles and embryos in mammals, thus *BMP4* is necessary for pregnancy success [21].

Many studies on *BMP4* gene divergence have been done in mammals, including cattle [22], sheep [23] and goats [24]. Ortiz et al. [25] investigated that the studied *BMP4* polymorphism in The G>T mutation at SNP rs109778173 was significantly associated (*p* < 0.01) with the number and ratio of possible cumulus-oophorus complexes, and the ratio of pregnancies at 30 days. Lari et al. [25], illustrated that the blastocyst rate was significantly linked with SNP rs109778173 of BMP4 (*p* = 0.006), but the relationship with the fertilization rate was not statistically significant (*p* = 0.095).

The objective of the present study to examine whether leptin (*LEP*), leptin receptor (*LEPR*), and *BMP4* genes could be a determining factor for the incidence of repeat breeder in Egyptian buffaloes.

## Methods

### Experimental animals and samples preparation

This study was carried out at Animal Production Research Institute, Agricultural Research Center, and Ministry of Agriculture. The field study was carried out at Animal Production Experimental Stations, (Mehallet Moussa, Alnataf Alqadim, and Alnataf Aljadid) Kafer El-Sheikh Governorate (located in the north of the Nile Delta). A total of 160 female animals (heifers and buffaloes, naturally inseminated) were recorded, 108 were fertile and 52 were repeat breeders (raised during two consecutive years 2019 and 2020).

### Survey study

Animals were kept under the regular systems of feeding and management adopted by the Animal Production Research Institute. Fresh water was available at all times. Buffaloes were housed in semi-open sheds.

### Diagnostic study

During this study, animals were observed visually for oestrus activity in presence of a teaser buffalo bull all the time. Length, signs, and duration of oestrus were individually recorded. Animals in heat were served using a fertile buffalo bull.The examination of reproductive tract of all animals by rectal palpation and ultrasonography revealed that the genitalia of all animals were free from any pathological diseases and disorders.

Also, interval from introducing buffalo bulls to incidence of 1st oestrus was recorded. The animals conceiving after the 1st service were chosen as normal animals. Animals failed to conceive after the 1st service were observed for the following oestrus activity and served at the 2nd oestrus. Animals failed to conceive after the 2nd service were observed for the following oestrus and served at the 3rd oestrus. Animals that failed to conceive after the 3rd service were considered as repeat breeder.

### Genomic DNA extraction and PCR amplification

DNA was extracted from the whole blood according to the method described by Miller et al. [26] with minor modifications. Briefly, blood samples were mixed with cold 2× sucrose-triton and centrifuged at 5,000 rpm for 15 min at 4°C. The nuclear pellet was suspended in lysis buffer, sodium dodecyl sulfate and proteinase K and incubated overnight in a shaking water bath at 37 °C. Nucleic acids were extracted with saturated NaCl solution. The DNA was picked up and washed in 70% ethanol. The DNA was dissolved in 1× TE buffer. The DNA concentration of each sample was estimated via NanoDrop 1000 (Thermo Scientific, UK), then adjusted to concentration of 50 ng/μL. Two (2μl) of buffalo DNA (50 ng/μL) was added as template into the PCR mixture (~ 23 μl) (Fermentas, Germany), which consists of 2 μl of dNTPs (2.5 mM each), 2 μl of PCR Buffer (10×), 0.5 μl TaqTM DNA polymerase (5 μ/ml), 1 μl forward primer (10 pmol), 1 μl reverse primer (10 pmol) and 12.5 μl sterilized distilled water in a 0.5-ml microfuge tube. Thermal cycling conditions were as follows: 94 °C for 5 min; 35 cycle of 94 °C for 45 s, annealing at specific temperature for each tested gene (*LEP, LEPR, BMP4*) for 45 s, and extension at 72 °C for 45 s followed by a final step of 72 °C for 5 min (Table 1). Then, the samples were analyzed by agarose gel electrophoresis using a DNA molecular weight marker (Fermentas, Germany). Then, PCR products were indicated by electrophoresis on 2% agarose gel, stained with ethidium bromide and visualized with the Gel Documentation System (BioRad, USA).

**Table 1:** List of primers sequences and restriction enzymes

Gene name	Studied part	Primers	Annealing temp	Characterization	Reference
**Leptin**	Exon 2 partial sequence	ATGCGCTGTGGACCCCTGTATC TGGTGTCATCCTGGACCTTCC	52°C	RFLP*-Kpn2I*	*Buchanan et al.* [27]
**Leptin receptor**	Exon 2	ACTACAGATGCTCTACTTTGG TGCTCCTCCTCAGTTT	51°C	sequence	*Almeida et al.* [28]
***BMP4***	Intron 5 and exon 6	TGGAACAGGAGAATGAGATAT TTATTTTGCGAATCCTGAGT	55°C	RFLP*-Hinf1*	*Ortiz et al.* [24]

### PCR-RFLP genotyping

*LEP* and *BMP4* amplicons were subjected to RFLP by *Kpn2I* and *Hinf1* digestion, respectively, while *LEPR* products were sequenced. Each digestion reaction (15 μL) involved 5 μl PCR product, 0.5 μl restriction enzyme (Promega, USA), 8.5 μL dH2O, and 1 μL buffer B 10× (Promega, USA). Incubation was performed at 37 °C for 10 min. Followed by agarose gel (2%) electrophoresis and visualization by Gel Documentation System (BioRad, USA).

### DNA sequencing and statistical data analysis

PCR products of normal and repeat breeder animals with different patterns attained by RFLP analysis were sequenced. The PCR bands with expected size were purified with the PCR purification kit (Qiagen, Germany). Purified amplicons were sequenced by Macrogen Incorporation (Seoul, South Korea). Sequence analysis and alignment was carried out using NCBI/BLAST/blastn suite to identify the SNPs among different patterns and alleles.

Sequencing data were compared against the GenBank database using Basic Local Alignment Search Tool (BLAST) tool [29], to identify the homology between target regions and river buffalo (*Bubalus bubalis*) records in the GenBank database.

To predict the effects of the non-synonymous SNPs on protein functions, the consensus classifier software PredictSNP was used which combined the results of MAPP, PhD-SNP, PolyPhen-1, PolyPhen-2, SIFT, and SNAP softwares [30]. The conservation score of the amino acids within the target proteins was computed using ConSurf server [31].

## Results

### Genotyping and sequencing results of LEP amplicon

**Fig. 1:**
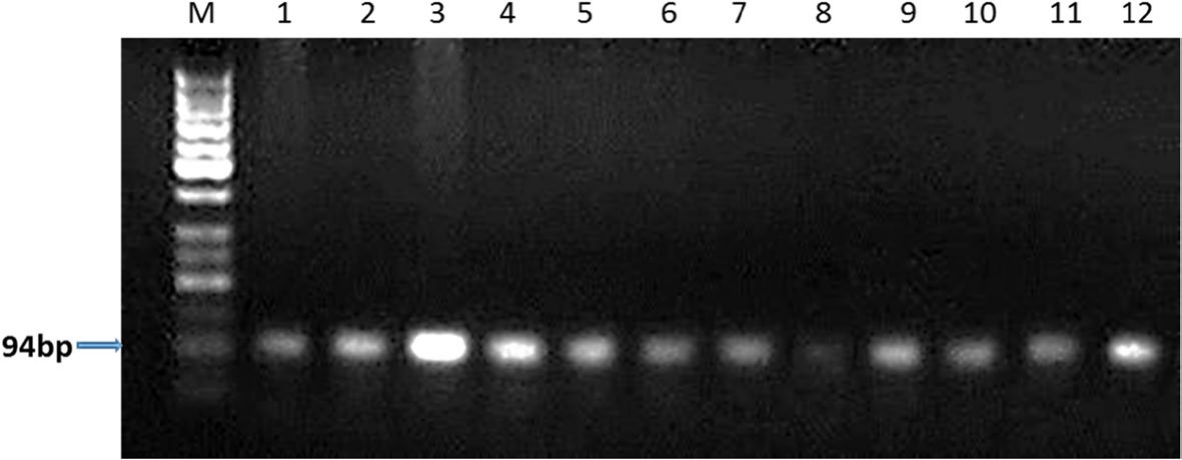
PCR product of *LEP* gene, M: 50 bp ladder, lanes 1–12: fertile and repeat breeder buffaloes

**Fig. 2:**
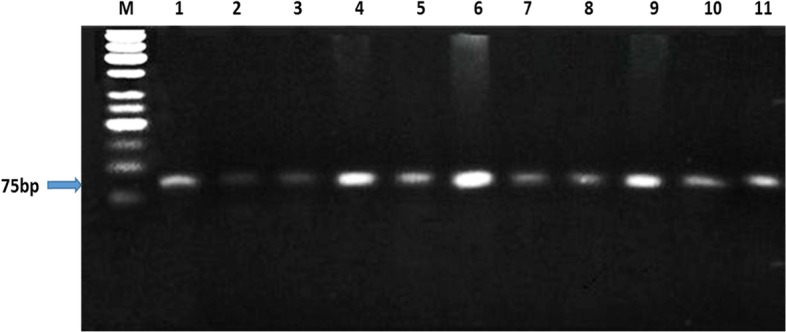
*LEP-Kpn2I*/PCR-RFLP, M: 50 bp ladder, lanes 1–11: fertile and repeat breeder buffaloes

The PCR product size of the amplified Egyptian river buffalo *LEP* gene fragment was 94 bp which covers a part of exon2 (Fig. 1).The RFLP analysis revealed single type of banding pattern yielding two fragments, of 75 bp and 19 bp (Fig. 2). The monomorphic banding patterns of the digested product revealed that all the animals (fertile and repeat breeder Egyptian buffaloes) possessed similar genotype (CC).

The sequence of the amplified region from the *LEP* gene was found to have this nucleotide sequence:

ATGCGCTGTGGACCCCTGTACCAATTCCTGTGGCTTTGGCCCTATCTGTCCTACGTGGAGGCTGTGCCCATCCGGAAGGTCCAGGATGACACCA

The amplified region from exon 2 consists of a part from the gene-coding region. Moreover, the first 63 nucleotides code for 21 amino acids represent the signal peptides of leptin polypeptide (Figure S1). The sequencing results confirmed the RFLP results that all the samples are monomorphic (CC genotype) and had the *Kpn2I* restriction site.

**Fig. 3:**
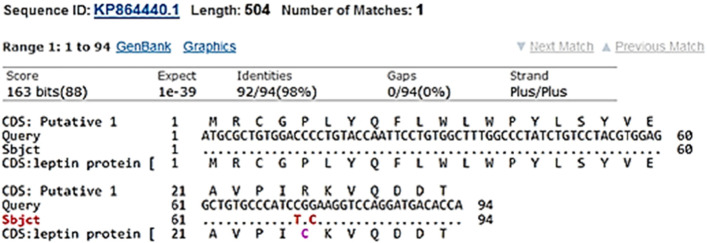
Detected C73T and G75T SNPs in the amplified *LEP* sequence and its homologues *Bubalus bubalis* sequence in the GenBank database

The sequence of the amplicon which performed in this study was compared with other buffalo breeds records in GenBank. Two SNPs (C73T and G75C) were detected between the sequenced sequences in our study and GenBank record KP864440 (Fig. 3) All our samples were monomorphic for these SNPs since all the sequenced samples had C and G alleles in the positions 73 and 75, respectively, as shown in Figures S2 and S3. Moreover, GenBank record KP864440 had T and C alleles in the positions 73 and 75, respectively. The C73T SNP was found to be non-synonymous SNP which caused R to C replacement the position 25 within the leptin polypeptide (position 4 in the mature form; R4C). On the other hand, G75C a synonymous SNP and did not change the amino acid sequence.

The non-synonymous SNP in *LEP* gene were evaluated by Predict SNP, which combined the results of several programs which utilize different methods to predict the deleterious effect of the non-synonymous SNP. R25C mutation was classified as a deleterious mutation using Predict SNP with a low expected accuracy (61%). The target mutation was classified as deleterious mutation by MAPP POLYPHEN-1, POLYPHEN-2, and SIFT softwares while both of PHD-SNP and SNAP tools were classified it as a neutral mutation (Figure S3). Notably, the amino acids R25 had the lowest conservation score which supports the R25C mutation is a neutral mutation, which may not affect function or structure of *LEP* protein.

### Sequencing results of LEPR amplicon

**Fig. 4:**
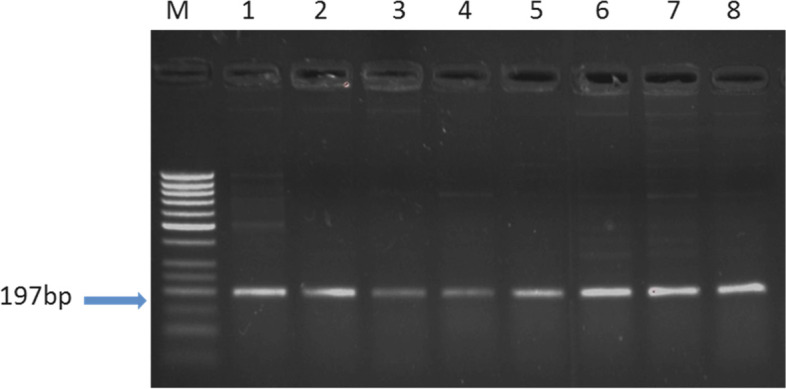
PCR product of *LEPR* gene, M: 50bp ladder, lanes 1–8: fertile and repeat breeder buffaloes

The molecular size of the PCR amplified products was estimated to be 197 bp for *LEPR* using a mutated forward primer and normal reverse primer comparing with DNA size markers and sequencing (Fig. 4).

The sequence of the amplified region from the *LEPR* gene was found to have this nucleotide sequence:

ACTACAGATGCTCTACTTTTGACGACTCCAGATCTTGAAAAGGGTTCTATTGGTATTAGTGACCAATGCAGCAGTGCTCAATTCTCTGAGGTTGAAAGCACAGACATAACCTGTGAGGATGAGAGCAGGAGACAGCCCTCTGTTAAATATGCCACCCTGCTCAGCAACTCTAAATCAGGTGAAACTGAGGAGGAGCA

Sequencing of the amplified region of the Egyptian buffalo leptin receptor gene covers a part of exon 2.The amplified region from exon 2 consists of a part from the gene coding region (Figure S4).The sequencing results showed that all the tested samples were monomorphic (CC genotype).

**Fig. 5:**
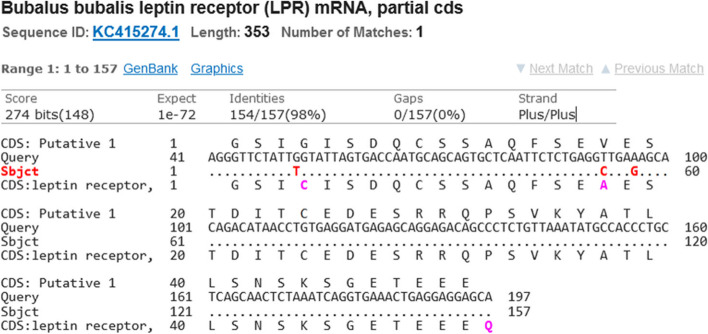
The detected G52T, T92C and A96G SNPs in the amplified *LEPR* sequence and its homologues *Bubalus bubalis* sequence in the GenBank database

Blasting of the sequenced DNA sequences to river buffaloes GenBank records showed one synonymous SNP (A96G) and 2 non-synonymous SNPs (G52T and T92C) compared to the GenBank record KC415274.1 (Fig. 5). The G52T and T92C SNPs lead to G954C and V967A replacement, respectively, in the *LEPR* amino acid sequence.

The Predict SNP results (Fig. S5) displayed that all the programs categorized both of G954C and V967A substitutions as neutral mutations with a total Predict SNP expected accuracy 87%. Evaluation of the amino acid conservativity depending on *LEPR* amino acids sequences belongs to many species showed that the amino acid G954 had slight conservation scale while V967 is less conserved and had average conservation scale. The relative conservation degrees may support the classification of the 2 substitutions as neutral mutations using PredictSNP tool.

### Genotyping and sequencing results of BMP4 amplicon

**Fig. 6:**
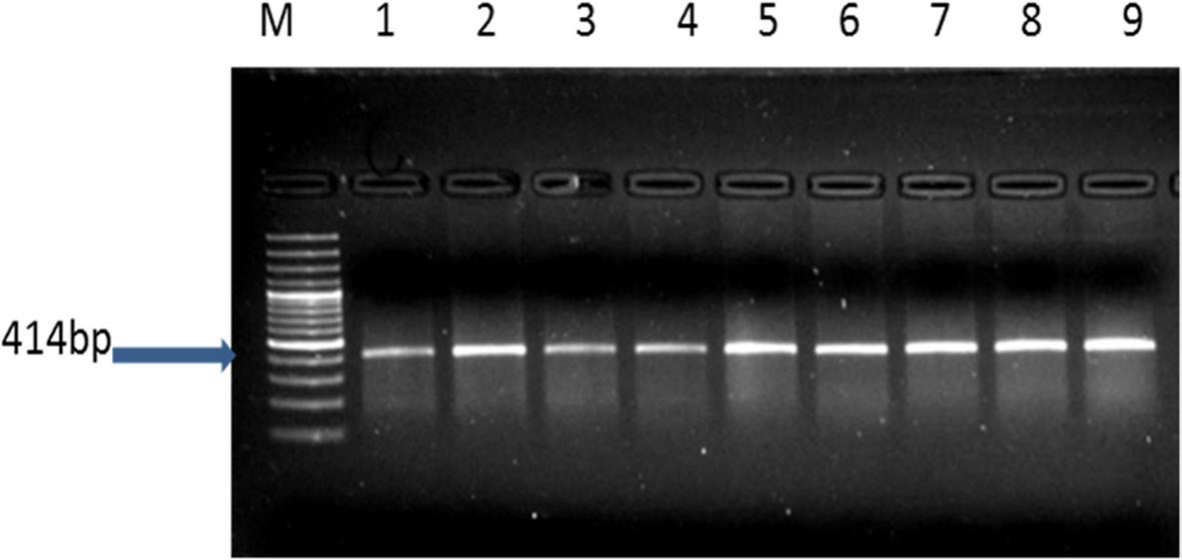
PCR product of *BMP4* gene, M: 100 bp ladder, lanes 1–9: fertile and repeat breeder buffaloes

**Fig. 7:**
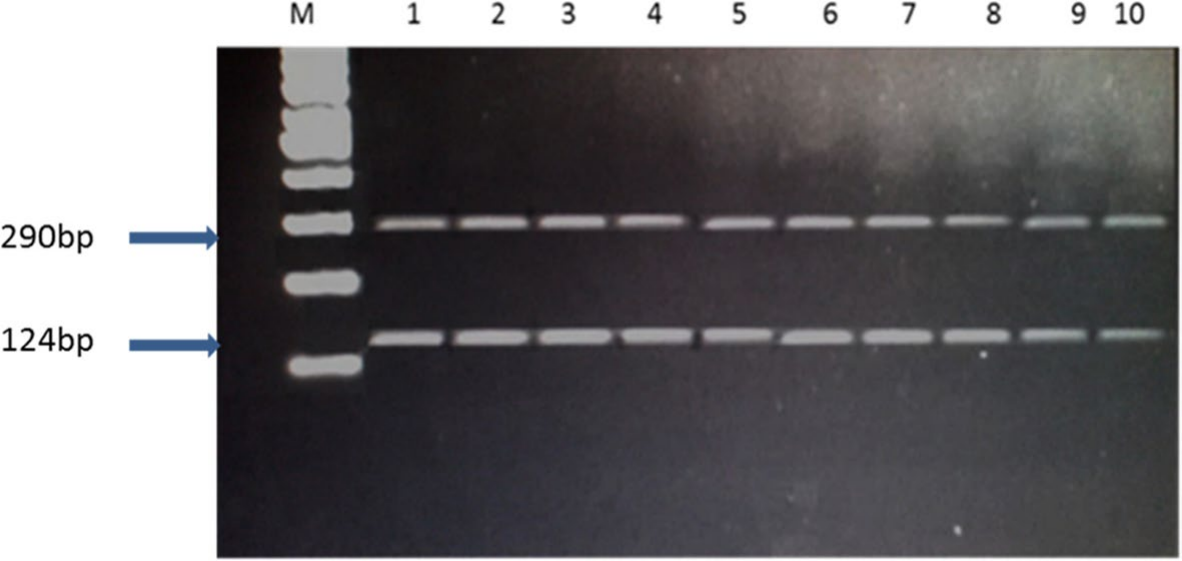
PCR-RFLP fragment of *BMP4* gene, M: 100 bp ladder, lanes 1–10: fertile and repeat breeder buffaloes

The PCR product size of the amplified Egyptian river buffalo *BMP4* gene fragment was 414 bp (Fig. 6). The digestion results of amplified fragment using restriction enzyme *Hinf1* was observed as 124 and 290 bp bands indicated GG genotype in all tested samples (Fig. 7).

The sequence of the amplified region from the *BMP4* gene was found to have this nucleotide sequence:

CCCCAATGGTGCCTAGAACATCTGGAGAACATCCCAGGGACCAGCGAAAACTCTGCTTTTCGTTTCCTCTTTAACCTCAGCAGCATCCCAGAGAACGAGGTGATCTCGTCTGCCGAGCTTCGACTCTTCCGGGAGCAGGTGGACCAGGGCCCTGACTGGGAGCAGGGCTTTCATCGTATAAACATTTATGAGGTTATGAAGCCCCCGGCAGAAGTGGTGCCTGGGCACCTCATCACACGACTACTGGACACAAGACTGGTCCACCACAATGTGACGCGGTGGGAAACTTTTGATGTGAGCCCTGCAGTCCTTCGCTGGACCCGGGAGAAGCAGCCCAACTATGGGCTGGCCATTGAGGTGACCCACCTCCATCAGACACGGACCCACCAGGGCCAGCATGTCAGGATTAGCCGA

Sequencing of the amplified region of the Egyptian buffalo *BMP4* gene produced a nucleotide sequence of 414 bp covers a part of intron 5 (1–16 bp) and exon 6 (17–414 bp). The amplified region from exon 6 consists of a part from the gene coding region (17–414 bp; Figure S6). No SNPs were found within the amplified region. This pattern referred to fixed GG genotype. The sequencing results shows *HinfI* restriction site mentioned to fixed GG genotype (CC genotype in the anti-sense strand) in the all sequenced samples (Figure S7).

## Discussion

All livestock producers strive to improve production qualities and increase genetic and economic gain. Traditional selection techniques or breeding programs employing genetic markers can help accomplish this gain. Analyses of increased genetic gain utilizing marker assisted selection can more precisely place genetic evaluation and reduce the time required for realization in livestock breeding [15].

The candidate gene strategy, which has been proposed as a direct search for Quantitative Trait Loci (QTL) to improve quantitative traits, is a good breeding tool that can increase the frequency of multiple births early in life. Detection of genetic markers, as well as mutants of genes linked to economically relevant features, could help breeders to devise feasible animal breeding programs [20].

Because the leptin gene is linked to productive and reproductive features both directly and indirectly, it's crucial to look into molecular markers that can look for connections with economically important traits [32].

The results of this study revealed that RFLP and sequencing results of leptin gene were monomorphic (CC) genotype in fertile and repeat breeder female buffaloes and the C73T SNP was found to be non-synonymous which caused R to C replacement in the position 25, a neutral mutation , within the leptin polypeptide. Similarly, in cattle Hilmia et al. [33] recorded three SNP on exon 2 leptin gene one synonymous SNP (S17S) and two non-synonymous SNPs which changed the amino acid encoding (R25C, R25H) and found that C allele was higher than A and T allele. Which in line with the results of Liefers et al. [34] who indicated that the SNP R25C is a non-synonymous mutation that can change the biological function of leptin gene. In cattle, de Oliviera et al. [35] reported that C to T SNP leads to non-conserved R to C replacement in position 4 of the mature leptin protein. This mutation is common, with allele frequencies ranging between 0.59 and 0.41, and the amino acid position 4 differs greatly between species. R4C substitution is not functional and this position may be polymorphic in other species. Another study on the polymorphism in the leptin gene in bovine found that a C to T SNP leads to R to C substitution at amino acid 4, which located within the non-conserved region of the A-helix in the mature leptin molecule. The R4C replacement leads to an unpaired cysteine to the mature leptin protein which may not affect its tertiary structure and function [36].

On the other hand, Fernandes et al. [32] revealed that, for the C305T SNP, there was a prevalence of the C allele (82%) over the T allele (0.18) and higher frequency for the CC genotype (0.67) in heifers. In a study with taurine breed, Buchanan et al. [37] observed rates of 0.54 and 0.46 for the C and T alleles, respectively, and these authors also discovered a greater frequency for the C allele. Although, other studies by Lagonigro et al. [27]; Liefers et al. [36] showed that in exon 2, the leptin SNPs C305T and A252T contribute amino acids alterations (Arg to Cys at position 305 and Tyr to Phe at position 252, respectively), which may affect the mature protein's tertiary structure. In beef heifers, Almeida et al. [38] discovered a strong link between another leptin SNP, RFLP1, and both weight at first calving and subsequent calving interval. Oikonomou et al. [28] evaluated the association between leptin SNP and many reproductive parameters, but only found a minor increase in the frequency of metritis with a SNP placed in intron 2 in a herd of Greek dairy cows. In Holstein cows, Clempson et al. [13] reported that the majority of the SNPs A59V (CC genotype) studied had substantial relationships with crucial reproductive variables including age at first service.

According to our study the sequences results of *LEPR* gene showed that the amplified fragment samples were monomorphic (CC genotype). Blasting of the DNA sequences to river buffaloes GenBank records showed one synonymous SNP (T102C) and 2 non-synonymous SNPs (A106G and C146A). The A106G and C146A SNPs lead to V967A and G954C replacement, respectively. Chen et al. [39] reported that, in Porcine, there were strong relationships between intron 2 and exons 2 and 18 polymorphisms and reproductive features. As well as Alim et al. [40] revealed that two SNPs detected within intron 3 and one SNP in exon 4 of *LEPR* gene and goats with heterozygous genotype AG at the loci g.104911A>G and g.105151A>G showed the highest prolificacy performance when compared with the other, homozygous genotypes. Sun et al. [41] investigated that the majority of studied features in Luchuan and Large White pigs with AA genotype were greater than those with AB and BB genotypes (*P* 0.05), according to the association analysis in the analyzed locus of *LEPR* gene exon 2 and in terms of litter size attributes, the AA genotype outperformed the AB and BB genotypes. Liefers et al. [42] found that throughout late pregnancy, animals with genotype CC had considerably greater circulating leptin concentrations than those with genotype CT, but there was no association during lactation in Holstein Friesian cows.

In Holstein-Friesian cattle, Komisarek [43] found that daughters of bulls with the TC genotype at T945M of the LEPR gene had a younger age of first insemination than daughters of CC homozygotes. Likewise, Trakovická et al. [15] investigated the relationship between SNP *LEPR*/T945M genotypes and a number of reproductive parameters, including age at first calving, calving interval, days open, and insemination interval. Only the calving interval length revealed significant differences (*P* 0.01). Cows with the heterozygous *LEPR*/T945MCT genotype have a shorter calving interval. This is in accord with the findings of Banos et al. [44], who also found no significant associations with milk production, feed intake, or body energy traits in UK dairy cows. Despite this, the LEPR plays a critical role in the activation of several downstream signaling pathways including the janus kinases/signal transducer and activation of transcription (JAK/STAT) pathway [45] to influence NPY expression (Neuropeptide Y), cell proliferation, and cell survival [46].

The amplified region of the Egyptian river buffalo *BMP4* gene represented a 414 bp PCR product. The *HinfI* digested PCR product had a consistent PCR-RFLP pattern with 124 and 290 bp bands. This pattern was confirmed by direct sequencing data, which indicated a fixed GG genotype (which corresponds to a CC genotype in the sense strand) in all animals, as well as a *HinfI* restriction site. While TT or TG genotypes was not detected in this study, the TT and TG genotypes frequency in Holstein cows were 5.37 and 37.85%, respectively [47], and 32 and 4% in Gyr cows [24].

Similar to this finding, Sharma et al. [48] identified an SNP (G1534A) in exon 2 and a microsatellite in 3′ flanking region of *BMP4* gene in nine different goat breeds of India. In a study on Small Tail Han sheep, Chu et al. [49] also reported one single nucleotide mutation C→A at 305 bp of exon 3 of *BMP4* gene in genotype BB in comparison with genotype AA and found that genotype BB had 0.61 or 1.01 lambs more than those with genotype AB or AA. Polymorphism in the *BMP4* gene of goats was originally described by Fang et al. [50]. They found no polymorphisms in the coding area of three different goat breeds raised in China, but two novel SNPs in the intronic region (EU104684: g.1986ANG, 2203GNA) were discovered. Also, Chu et al. [51] used single strand conformation polymorphism to find SNPs in exon 2 and intron 2 of the *BMP4* gene in both high fecundity (Jining Grey goat) and low fecundity (Boer, Angora, and Inner Mongolia Cashmere goats).

## Conclusion

In this study, genotyping and sequencing of *LEP*, *LEPR*, and *BMP4* afforded no association between the ascertained mutations and the incidence of repeat breeding in Egyptian buffaloes suggesting no clear-cut evidence that warrant genetic divergence.

## Supplementary Information

**Additional file 1: Figure S1.** The nucleotides and translated amino acids sequences of amplified fragment from the Egyptian river buffalo *LEP* gene (Exon 2 partial sequences). CDS: Coding Sequence. **Figure S2.** A chromatogram showing *Kpn2I* restriction site within the amplified fragment of the bubaline *LEP* gene. **Figure S3.** In silico prediction of the effect of the target non-synonymous SNP on river buffalo *LEP* function. A Evaluation of the effect of R25C replacement using Predict SNP tool. B The conservation degree of the amino acid R25 within *LEP* polypeptide. **Figure S4.** The nucleotides and translated amino acids sequences of amplified fragment from the Egyptian river buffalo *LEPR* (Exon 2 partial sequences). CDS: Coding Sequence. **Figure S5.** In silico prediction of the effect of the target non-synonymous SNPs on river buffalo *LEPR* function. A) Evaluation of the effect of the G954C and V967A replacements using PredictSNP tool. B) The conservation degree of the amino acids G954 and V967 within *LEPR* polypeptide. **Figure S6.** The nucleotides and translated amino acids sequences of amplified fragment from the Egyptian river buffalo *BMP4* (intron 5 and Exon 6 partial sequences). CDS: Coding Sequence. **Figure S7.** A part of *BMP4* fragment sequencing chromatogram shows *HinfI* restriction site.

## Data Availability

All data generated or analyzed during this study are included in this published article.
