# Supplementary material for: Genetic structure of some candidate genes of repeat breeder syndrome in Egyptian buffaloes
Source: J Genet Eng Biotechnol. 2022 Jul 22;20:110. doi: 10.1186/s43141-022-00397-2 (PMC9307700; doi:10.1186/s43141-022-00397-2)
Supplement: Supplementary file 1 — Additional file 1: Figure S1. The nucleotides and translated amino acids sequences of amplified fragment from the Egyptian river buffalo LEP gene (Exon 2 partial sequences). CDS: Coding Sequence. Figure S2. A chromatogram showing Kpn2I restriction site within the amplified fragment of the bubaline LEP gene. Figure S3. In silico prediction of the effect of the target non-synonymous SNP on river buffalo LEP function. A Evaluation of the effect of R25C replacement using Predict SNP tool. B The conservation degree of the amino acid R25 within LEP polypeptide. Figure S4. The nucleotides and translated amino acids sequences of amplified fragment from the Egyptian river buffalo LEPR (Exon 2 partial sequences). CDS: Coding Sequence. Figure S5. In silico prediction of the effect of the target non-synonymous SNPs on river buffalo LEPR function. A) Evaluation of the effect of the G954C and V967A replacements using PredictSNP tool. B) The conservation degree of the amino acids G954 and V967 within LEPR polypeptide. Figure S6. The nucleotides and translated amino acids sequences of amplified fragment from the Egyptian river buffalo BMP4 (intron 5 and Exon 6 partial sequences). CDS: Coding Sequence. Figure S7. A part of BMP4 fragment sequencing chromatogram shows HinfI restriction site. [file 43141_2022_397_MOESM1_ESM.docx]

**
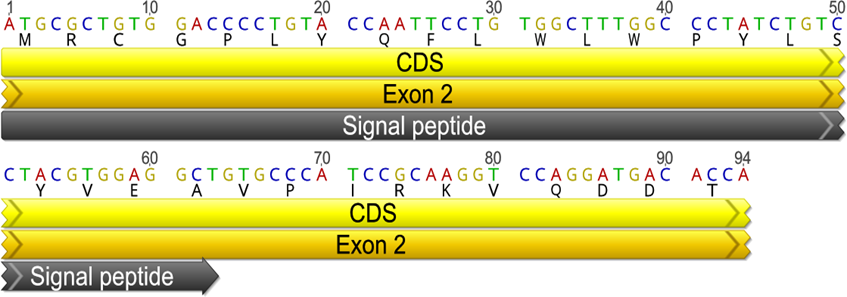
Figure S1`:** The nucleotides and translated amino acids sequences of amplified fragment from the Egyptian river buffalo *LEP* gene (Exon 2 partial sequences). CDS: Coding Sequence.


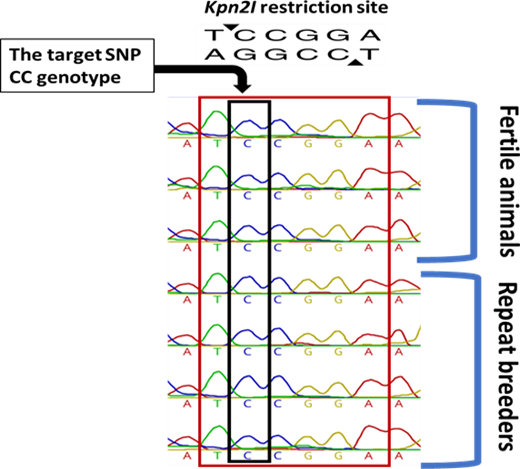


**Figure S2:** A chromatogram showing *Kpn2I* restriction site within the amplified fragment of the bubaline *LEP* gene.


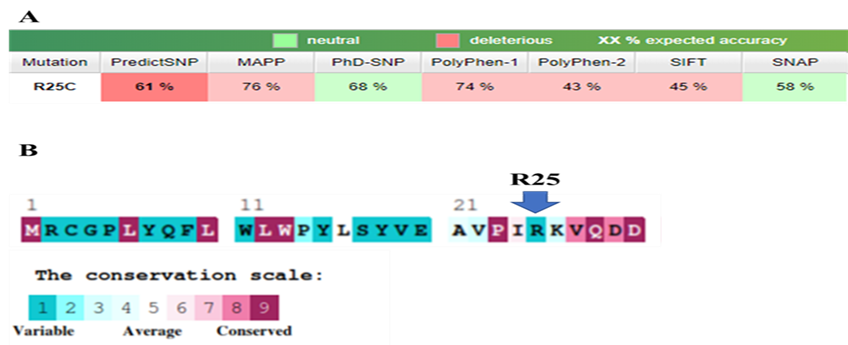


**Figure S3:** In silico prediction of the effect of the target non-synonymous SNP on river buffalo *LEP* function.

A) Evaluation of the effect of R25C replacement using Predict SNP tool.

B) The conservation degree of the amino acid R25 within *LEP* polypeptide.


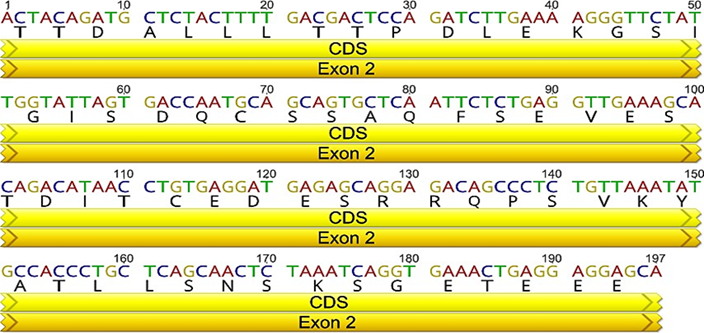


**Figure S4:** The nucleotides and translated amino acids sequences of amplified fragment from the Egyptian river buffalo *LEPR* (Exon 2 partial sequences). CDS: Coding Sequence.


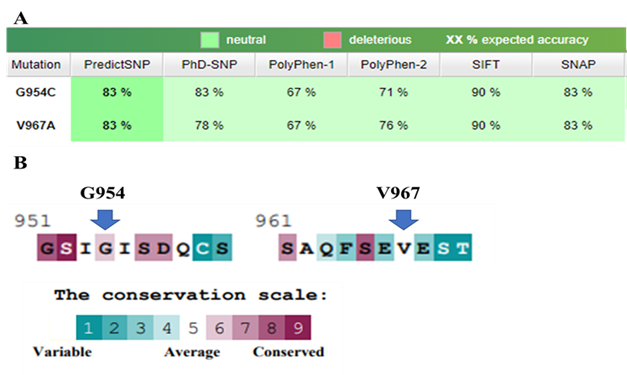


**Figure S5:** In silico prediction of the effect of the target non-synonymous SNPs on river buffalo *LEPR* function. A) Evaluation of the effect of the G954C and V967A replacements using PredictSNP tool. B) The conservation degree of the amino acids G954 and V967 within *LEPR* polypeptide.


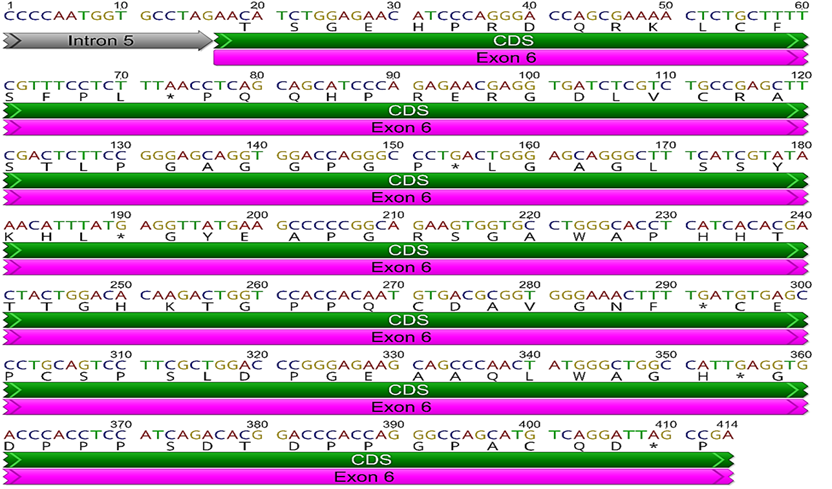


**Figure S6:** The nucleotides and translated amino acids sequences of amplified fragment from the Egyptian river buffalo *BMP4* (intron 5 and Exon 6 partial sequences). CDS: Coding Sequence.


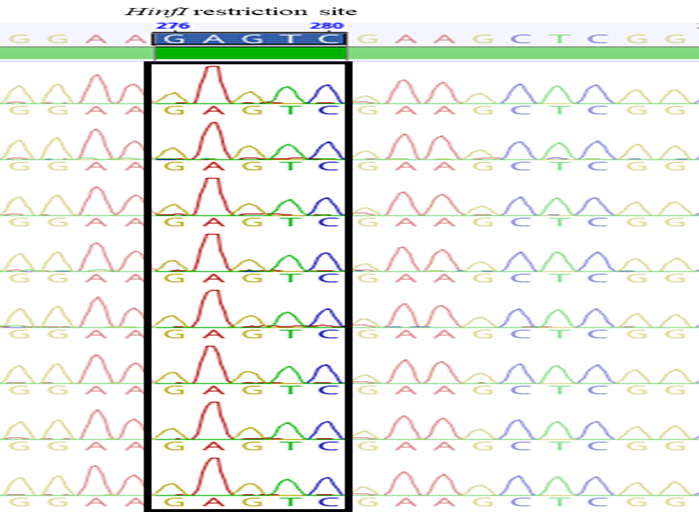


**Figure S7:** A part of *BMP4* fragment sequencing chromatogram shows *HinfI* restriction site.
